# Hyperosmolar hyperglycemic state with severe hypernatremia coexisting with central diabetes insipidus: A case report and literature review

**DOI:** 10.1515/biol-2025-1194

**Published:** 2025-11-06

**Authors:** Congcong Yao, Lishuang Zhu, Songtao Shou, Heng Jin, Yan Zhang

**Affiliations:** Department of Emergency Medicine, Tianjin Medical University General Hospital, 154 Anshan Road, Heping District, Tianjin, 300052, China; Department of Critical Care Medicine, Tianjin Medical University General Hospital, 154 Anshan Road, Heping District, Tianjin, 300052, China

**Keywords:** central diabetes insipidus, hyperosmolar hyperglycemic state, type 1 diabetes mellitus, hypernatremia, ventricular fibrillation

## Abstract

Diabetes insipidus is characterized by polyuria and polydipsia, often resulting from central or nephrogenic causes. In diabetic emergencies, hyperosmolar hyperglycemic state (HHS), severe hypernatremia, and ventricular fibrillation are life-threatening conditions that require prompt intervention. This report describes a 47-year-old male with poorly controlled diabetes mellitus, who developed coma, excessive thirst, polyuria, hyperglycemia (47.29 mmol/L), hypernatremia (195.6 mmol/L), and plasma hyperosmolality (385 mOsm/kg). Despite fluid resuscitation and insulin therapy, refractory hypernatremia persisted, leading to a diagnosis of central diabetes insipidus (CDI). The patient also developed ventricular fibrillation, which was managed with defibrillation. Concurrently, desmopressin and blood purification were administered to address CDI and severe hypernatremia. This case emphasizes the importance of considering CDI when polyuria persists despite glucose control. The occurrence of ventricular fibrillation underscores the necessity of continuous cardiac monitoring in the context of hypovolemia and severe electrolyte imbalance. We propose that diabetes mellitus-related vascular injury impairs blood flow in the hypothalamus–pituitary tract, disrupting arginine vasopressin synthesis and secretion, contributing to CDI in poorly controlled diabetes mellitus.

## Graphical abstract

**Figure:**
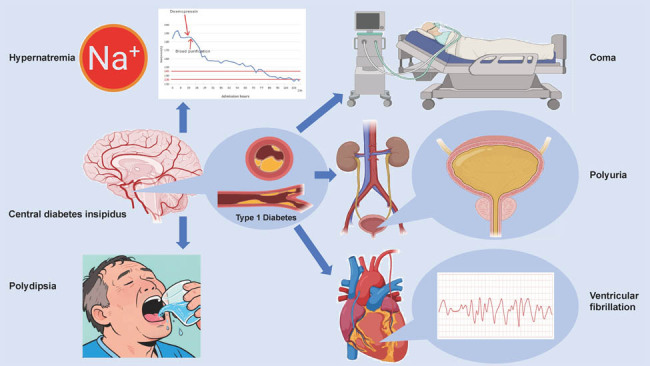

## 1 Introduction

Polyuria, defined as a daily urine output exceeding 3 L, is a common clinical manifestation observed in conditions such as diabetes mellitus, diabetes insipidus (DI), acute renal failure, and electrolyte imbalances [1]. In diabetic patients with well-controlled blood glucose levels who continue to experience persistent polyuria and polydipsia, the possibility of coexisting DI should be considered. DI, a component of the polyuria–polydipsia syndrome, is characterized by the excretion of large volumes of dilute urine accompanied by extreme thirst and polydipsia [2]. The differential diagnosis of polyuria involves distinguishing primary DI from secondary causes [3]. Central diabetes insipidus (CDI) results from reduced synthesis and secretion of arginine vasopressin (AVP) due to various etiologies, leading to markedly decreased plasma AVP levels [4]. Treatment with exogenous AVP is typically effective. Additionally, the measurement of copeptin – a stable surrogate marker for AVP secretion – serves as an important adjunctive tool in diagnosing CDI [5]. A markedly reduced plasma copeptin level indicates impaired synthesis or secretion of AVP by the hypothalamus or pituitary gland, thereby providing further confirmation of CDI [6]. CDI can be classified into primary and secondary forms [7]. Approximately 30% of cases are primary, often idiopathic or of unknown cause, while the remaining cases are secondary, arising from identifiable pathological processes: around 25% are associated with tumors in the hypothalamic–pituitary region, 16% follow traumatic brain injury, and approximately 20% occur as a complication of intracranial surgery [8].

Hyperosmolar hyperglycemic syndrome (HHS) is a severe and common acute complication of type 2 diabetes mellitus, with a reported mortality rate of up to 20% [9]. Due to its high lethality, HHS demands prompt recognition, accurate diagnosis, and aggressive management. Its onset is typically marked by classic diabetic symptoms, including excessive thirst, polydipsia, polyuria, and fatigue, which may be newly developed or exacerbated [10]. The hallmark clinical features of HHS include severe hyperglycemia, markedly elevated plasma osmolality, profound dehydration, and varying degrees of impaired consciousness [11,12]. The underlying pathophysiological mechanism is primarily attributed to a relative deficiency of insulin, which leads to excessive hyperglycemia. This, in turn, induces osmotic diuresis and significant fluid loss, ultimately culminating in a hyperosmolar state [13].

This case report describes a rare and clinically significant presentation of a 47-year-old male who initially manifested with coma, polyuria, and dehydration, the laboratory confirmed severe hypernatremia (180 mmol/L), followed by ventricular fibrillation. He was diagnosed with CDI in the setting of a hyperosmolar hyperglycemic coma – a complex and rarely documented clinical scenario. It is hypothesized that diabetes mellitus-related microvascular injury may impair the blood supply to the hypothalamic–pituitary axis, thereby disrupting the synthesis and secretion of AVP. This case exemplifies the complex interplay between metabolic dysregulation and neuroendocrine dysfunction, suggesting that chronic diabetes mellitus may serve as a precipitating factor for CDI – a diagnosis that is frequently overlooked.

## 2 Case presentation

### 2.1 Initial presentation and symptom onset

A 47-year-old male presented with a 19-day history of polydipsia and polyuria, followed by the onset of coma 1 day prior to admission. He has a 4-year history of unclassified diabetes, usually treated with oral Chinese medicine, with a maximum recorded blood glucose level of 47 mmol/L but had not been compliant with medication. Additionally, he had a 4-year history of hypertension, with a peak systolic blood pressure of 180 mmHg, also without regular treatment or monitoring. Nineteen days before presentation, the patient developed increased thirst, excessive fluid intake (approximately 5,000 mL/day), frequent urination including nocturia, and experienced a weight loss of 2.5 kg. He reported no fever, chills, nausea, vomiting, abdominal pain, diarrhea, visual disturbances, or altered mental status during this period, and did not seek medical care.


**Informed consent:** Informed consent has been obtained from all individuals included in this study.
**Ethical approval:** The research related to human use has been complied with all the relevant national regulations, institutional policies and in accordance with the tenets of the Helsinki Declaration, and has been approved by the Medical Ethics Committee of Tianjin Medical University General Hospital.

### 2.2 Initial emergency management at local hospital

One day before admission, he experienced a sudden loss of consciousness, became unresponsive, and developed respiratory difficulty. He was brought to Ninghe District Hospital in Tianjin, where arterial blood gas analysis revealed: pH 7.354, PO₂ 85.3 mmHg, PCO_2_ 21.9 mmHg, sodium 180 mmol/L, potassium 2.7 mmol/L, calcium 1.03 mmol/L, and lactate 1.5 mmol/L. A complete blood count showed: white blood cell count 25.17 × 10^9^/L, red blood cell count 6.12 × 10¹²/L, hemoglobin 179.0 g/L, and platelet count 288 × 10^9^/L. Biochemical tests revealed uric acid 1,255 μmol/L, glucose 47.29 mmol/L, creatinine 202 μmol/L, and urea 17.86 mmol/L.

### 2.3 Transfer and hospital admission

Despite treatment with 0.45% saline infusion, glucose-lowering therapy, and supportive care, the patient’s condition did not improve, prompting transfer to the Emergency Department of our hospital. Repeat blood gas analysis showed: pH 7.390, PCO₂ 45.5 mmHg, sodium 195.6 mmol/L, potassium 3.49 mmol/L, calcium 1.160 mmol/L, glucose 24.2 mmol/L, lactate 3.1 mmol/L, and plasma osmolality 385.0 mOsm/kg. Subsequent laboratory results indicated a white blood cell count of 14.14 × 10⁹/L, uric acid 938 μmol/L, creatine kinase 8,464 U/L, creatinine 118 μmol/L, and urea 8.0 mmol/L. Urinalysis revealed strongly positive glucose (++++), specific gravity of 1.014, negative ketones, and strongly positive hematuria (+++). Electrocardiography showed sinus rhythm. Additional test results are summarized in Table 1.

**Table 1:** Laboratory results of the patients

Laboratory test	Result	Reference value
Cortisol (μg/dL)	23.90	5.00–25.00
Adrenocorticotropic hormone (pg/mL)	20.80	0.00–46.00
Free triiodothyronine (pmol/L)	1.96	2.43–6.01
Free thyroxine (pmol/L)	7.12	9.01–19.05
Sensitive thyrotropin (μIU/mL)	0.640	0.350–4.940
Total cholesterol (mmol/L)	4.22	3.59–5.17
Triglycerides (mmol/L)	1.08	0.57–1.71
High-density lipoprotein cholesterol (mmol/L)	0.98	0.80–2.20
Low-density lipoprotein cholesterol (mmol/L)	2.41	1.33–3.36
Glycated hemoglobin (%)	16.40	4.00–6.00
Plasma aldosterone (ng/dL)	3.4	3.0–23.6
Plasma renin (μIU/mL)	11.6	2.8–39.9
Follicle-stimulating hormone (IU/L)	4.0	0.95–11.95
Luteinizing hormone (IU/L)	0.40	0.57–12.07
Prolactin (ng/mL)	4.36	3.46–19.40
Growth hormone (ng/mL)	5.67	0.06–4.940
Brain natriuretic peptide (pg/mL)	115	0.0–100.0
Troponin I (ng/mL)	<0.05	0.00–0.40
Myoglobin (ng/mL)	>500	0.0–107.0
Creatine kinase-MB isoenzyme (ng/mL)	14.0	0.0–4.3

### 2.4 Hospital admission and initial management

Within the first 24 h of admission, the patient underwent a comprehensive diagnostic evaluation, including measurements of plasma and urine osmolality, 24 h urinary electrolytes, urinary protein and glucose levels, as well as assessments of pituitary function (Table 1).

Treatment was initiated with a low-dose insulin infusion at a rate of 0.04 IU/kg/min for glycemic control, along with hypotonic fluid replacement using 0.45% saline. Additional supportive measures included acid suppression therapy, gastric mucosal protection, and correction of electrolyte imbalances. Over the 24 h period, a total of 5,375 mL of intravenous fluid was administered, while the recorded urine output was 5,620 mL.

### 2.5 Treatment adjustments

**Figure 1:**
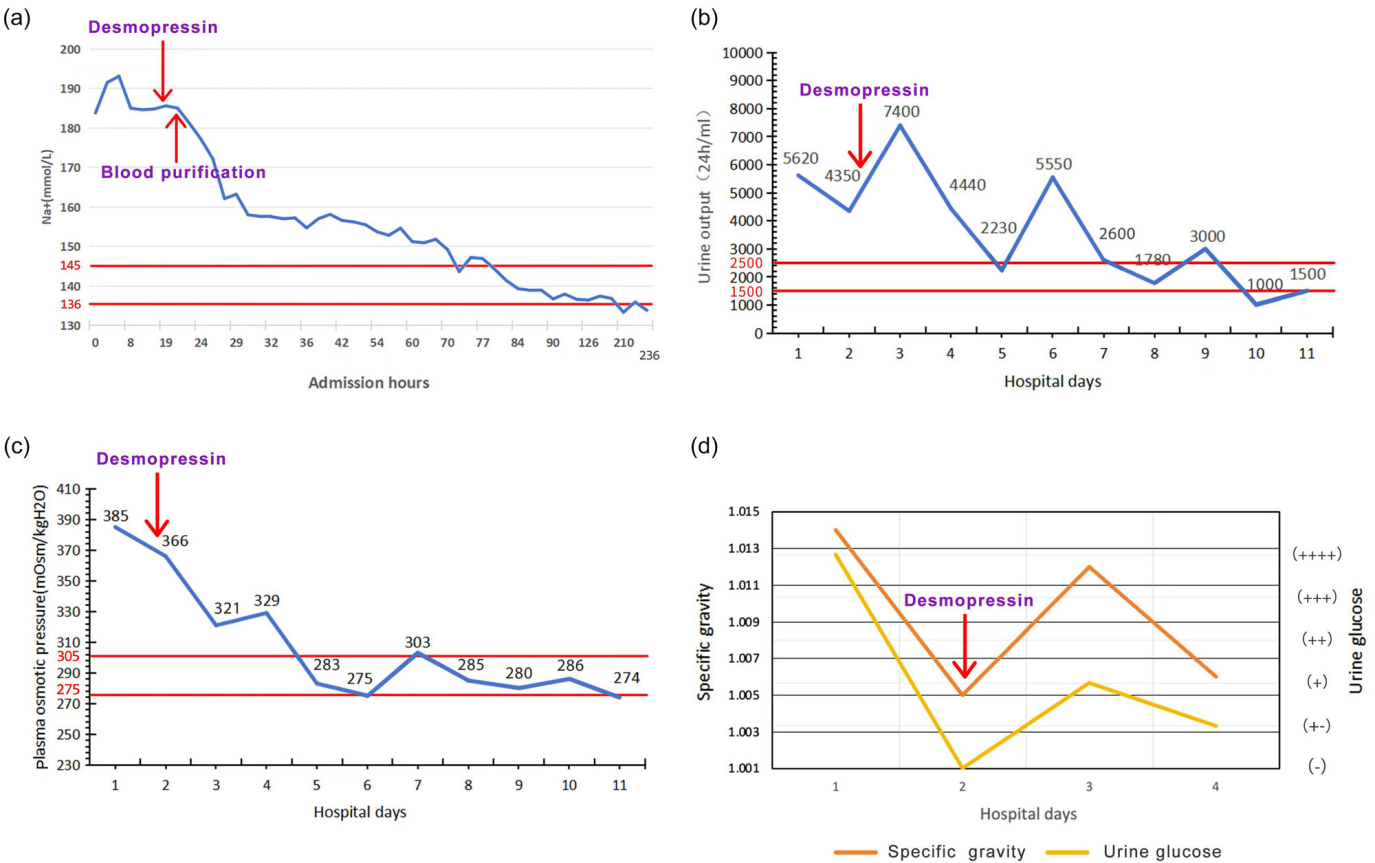
A graphical representation of changes in patients’ laboratory parameters. (a) Change trend of serum sodium after admission. (b) Change trend of urine specific gravity and urine glucose after admission. (c) Change trend of urine volume after admission. (d) Change trend of plasma osmolality after admission. Between the two red lines is the normal range. Specific gravity reference value: 1.005–1.030. Urine glucose reference value: (−). Vertical arrow indicates timing of initial desmopressin administration; maintenance therapy continued throughout hospitalization.

Following initial treatment, the patient’s blood glucose levels exhibited a downward trend; however, he remained comatose, hypernatremia persisted, and urine output did not significantly improve (Figure 1a–c). Urine specific gravity declined, and serum creatinine levels showed a decreasing trend. Serial measurements revealed urine specific gravity fluctuating between 1.014 and 1.005 (Figure 1d). The patient was diagnosed with HHS accompanied by hypernatremia upon admission. Patients with HHS typically present with a marked reduction in effective circulating blood volume, increased urine output due to osmotic diuresis, and elevated urine osmolality. However, in this case, despite glycemic control, the patient’s urine output did not significantly decrease and was accompanied by a decline in urine osmolality. Based on these findings, a deficiency in antidiuretic hormone was suspected, prompting the initiation of desmopressin therapy. Considering the possibility of CDI, the patient was treated with oral desmopressin acetate at a dose of 0.1 mg, three times daily.

During treatment, the patient suddenly developed ventricular tachycardia, which progressed to ventricular fibrillation, accompanied by a precipitous drop in blood pressure to 50/25 mmHg. Immediate cardiopulmonary resuscitation and defibrillation were performed. Arterial blood gas analysis at that time showed: pH 7.466, PCO₂ 42.8 mmHg, sodium 181.2 mmol/L, potassium 4.13 mmol/L, calcium 0.957 mmol/L, glucose 13.8 mmol/L, lactate 2.4 mmol/L, and plasma osmolality 365.00 mOsm/kg. The initial electrocardiographic indicated sinus rhythm. The episode of ventricular fibrillation was considered secondary to hypovolemia induced by severe hypernatremia. Desmopressin therapy was continued in combination with fluid resuscitation, glucose management, and intravenous lidocaine for antiarrhythmic treatment. The patient was intubated, placed on mechanical ventilation, and underwent bedside continuous blood purification to maintain internal homeostasis. The sodium concentration in the replacement fluid was adjusted to 128–133 mmol/L to gradually lower serum sodium levels.

Subsequently, the patient’s level of consciousness improved, accompanied by a downward trend in both serum sodium and urine output. After 38.5 h of continuous blood purification, serum sodium decreased to 151.8 mmol/L. Blood purification was then discontinued, while desmopressin therapy was maintained (oral desmopressin acetate at a dose of 0.1 mg, three times daily). Sodium levels normalized, urine output decreased to 750–2,500 mL/day, and symptoms of thirst, polydipsia, and dehydration resolved. Plasma osmolality decreased, and urine osmolality increased (Table 2). Oral glucose tolerance test (OGTT) and insulin release test were performed, along with pituitary magnetic resonance imaging (MRI). OGTT and insulin release test revealed the following results: plasma glucose levels were 12.10 mmol/L at 0 min, 14.70 mmol/L at 30 min, 16.88 mmol/L at 60 min, 24.73 mmol/L at 120 min, and 28.79 mmol/L at 180 min. Corresponding insulin levels were 1.00, 1.60, 2.00, 2.40, and 3.50 mIU/L, respectively. C-peptide levels were 0.61, 0.68, 0.72, 0.93, and 1.28 ng/mL, respectively. The patient was diagnosed with type 1 diabetes mellitus. Pituitary MRI demonstrated mildly heterogeneous signal intensity within the pituitary gland. The pituitary stalk was midline and of normal thickness. No evidence of a mass lesion, hemorrhage, or infarction was observed (Figure S1). Following the initiation of desmopressin, the patient’s urine output declined, and clinical symptoms improved, confirming the diagnosis of CDI in the context of HHS. Maintenance therapy included oral desmopressin (0.1 mg, three times daily) with daily urine output monitoring. Insulin therapy was also implemented: 12 IU of rapid-acting insulin (Rui Xiu Lin) before meals and 16 IU of long-acting insulin (Sanofi) at bedtime. The patient remained clinically stable, with postprandial blood glucose levels ranging from 7.5 to 9.0 mmol/L and urine output between 1,000 and 1,500 mL/day. He was discharged on hospital Day 19 and reported no discomfort at a 1-month follow-up. At the 3- and 6-month follow-ups, oral desmopressin acetate (0.1 mg, three times daily) was found to effectively control the symptoms of DI.

**Table 2:** Laboratory results before desmopressin (24 h post-admission) and after treatment (72 h post-desmopressin initiation)

Laboratory test	Before treatment	After treatment	Reference value
White blood cells (*10^9^/L)	14.14	6.69	3.50–9.50
Red blood cells (*10^12^/L)	4.89	3.56	4.30–5.80
Hemoglobin (g/L)	144	104	130–175
Platelets (*10^9^/L)	105	286	125–350
Prothrombin time (s)	12.2	12.2	9.5–15.0
Activated partial thromboplastin time (s)	26.8	30.0	20.0–40.0
Thrombin time (s)	25.5	15.2	13.0–25.0
Fibrinogen (g/L)	3.1	4.427	1.80–4.00
d-dimer (ng/mL)	377	950	0.00–500.0
Serum creatinine (μmol/L)	118	57	62–133
Uric acid (μmol/L)	938	192	140–414
Blood urea nitrogen (mmol/L)	8.0	4.2	2.5–7.1
Total protein (g/L)	51	54	65–85
Albumin (g/L)	27	27	40–55
Alanine aminotransferase (U/L)	37	23	9–50
Aspartate aminotransferase (U/L)	118	14	15–40
Total bilirubin (μmol/L)	9.1	8.3	≤26
pH	7.452	7.475	7.350–7.450
Na^+^ (mmol/L)	195.6	133.8	136.0–145.0
K^+^ (mmol/L)	2.93	3.93	3.50–5.10
PO_2_ (mmHg)	92.65	163.82	83.0–108.0
PCO_2_ (mmHg)	38.4	36.5	32.0–48.0
Glucose (mmol/L)	47.29	7.1	3.5–5.3
Lac (mmol/L)	2.4	1.0	0.6–1.4
Plasma osmotic pressure (mOsm/kgH_2_O)	385	303	275–305
Urine glucose	(++++)	(−)	(−)
Urine occult blood	(+++)	(−)	(−)
Urine pH	5.0	5.0	5.50–8.00
Urinary albumin	(+−)	(−)	(−)
Urine leukocyte esterase	(+++)	(−)	(−)

## 3 Discussion

The diagnosis of CDI in this case was established based on three key clinical criteria: (1) persistent hypotonic polyuria, as evidenced by a urine specific gravity of 1.005–1.014, despite severe hyperosmolality; (2) a marked reduction in urine output and an increase in urine osmolality following desmopressin administration (Table 2); and (3) absence of the posterior pituitary bright spot on MRI, a characteristic radiological indicator of CDI. We acknowledge that copeptin and AVP levels were not measured due to resource limitations and the patient’s critical condition. Additionally, the water deprivation test was contraindicated due to severe hypernatremia and unstable hemodynamic status. As such, the diagnosis of CDI was made based on clinical presentation (persistent polyuria, hypernatremia, low urine osmolality) and a positive clinical response to desmopressin, which rapidly reduced urine output and serum sodium levels. Nephrogenic diabetes insipidus (NDI) was excluded based on the observed response to desmopressin, as NDI typically shows resistance to vasopressin analogs.

This case highlights three critical principles in the management of complex metabolic emergencies: (1) Vigilance for dual pathology: in patients with HHS who present with persistent polyuria and refractory hypernatremia, CDI should be considered – particularly when polyuria persists despite normalization of blood glucose levels. Early administration of desmopressin reversed hypotonic polyuria and confirmed the diagnosis [14]. (2) Balanced electrolyte correction: rapid correction of hypernatremia (exceeding 0.5 mmol/L/h) increases the risk of cerebral edema, whereas delayed correction may exacerbate myocardial ischemia. In this case, bedside continuous renal replacement therapy using sodium-adjusted replacement fluid (128–133 mmol/L) enabled controlled serum sodium reduction while avoiding neurological complications. (3) Prevention of cardiac events: severe hypovolemia due to fluid loss from both HHS and CDI can compromise coronary perfusion. Concurrent electrolyte disturbances further reduce the threshold for life-threatening arrhythmias such as ventricular fibrillation. Continuous electrocardiographic monitoring and hemodynamic support are essential, with immediate defibrillation critical in the event of ventricular fibrillation.

Among the key insights, maintaining vigilance for dual pathology is particularly critical. In this case, the coexistence of HHS and CDI created a vicious cycle that significantly worsened the patient’s clinical condition. HHS, characterized by profound hyperglycemia, triggered osmotic diuresis, leading to severe dehydration and hypernatremia [11]. Concurrently, CDI – resulting from AVP deficiency – impaired renal water reabsorption, further exacerbating polyuria and hyperosmolality. The synergistic effect of HHS-induced osmotic diuresis and CDI-induced hypotonic polyuria caused profound volume depletion and hypernatremia, culminating in hypovolemic shock. This, in turn, compromised coronary perfusion and increased the risk of ventricular fibrillation, which ultimately occurred. Moreover, in patients with long-standing or poorly controlled diabetes mellitus, chronic hyperglycemia may cause microvascular lesions in the pituitary stalk, thereby impairing blood flow to AVP-producing neurons. This disruption can inhibit AVP synthesis and secretion, increasing susceptibility to CDI. Although rare, Wolfram syndrome – a genetic disorder characterized by the coexistence of DI, type 1 diabetes mellitus, optic atrophy, and deafness – should also be considered in such presentations [14,15]. However, the absence of visual or auditory abnormalities in this patient ruled out Wolfram syndrome. In conclusion, microvascular injury resulting from chronic hyperglycemia represents a plausible etiology for CDI in this case, although other possibilities, such as subclinical autoimmune hypophysitis, warrant consideration. While pituitary MRI revealed no structural abnormalities, diabetic microangiopathy could have caused pituitary stalk ischemia, impairing AVP axonal transport.

This pathophysiological hypothesis requires further validation in future studies. Clinically, the focus should remain on recognizing the pathological interplay between HHS and CDI in the context of uncontrolled type 1 diabetes mellitus.

DI is characterized by impaired renal water reabsorption, either due to a deficiency of AVP or renal resistance to its action, resulting in hypotonic polyuria and polydipsia [16]. It is a rare disorder, with an estimated prevalence of approximately 1 in 25,000 individuals, affecting all age groups and occurring equally across genders [17,18]. The pathophysiology of DI involves disruptions in the synthesis or transport of AVP by the supraoptic and paraventricular nuclei of the hypothalamus. This results in an inability of the kidneys to reabsorb water in the collecting ducts, leading to the excretion of large volumes of dilute urine, increased plasma osmolality, and stimulation of the hypothalamic thirst center, thereby causing excessive thirst and fluid intake [19]. CDI arises from AVP deficiency due to dysfunction of the hypothalamic–pituitary axis. Etiologies of CDI can be either acquired or hereditary [20]. Acquired CDI is more common and typically occurs when more than 80% of AVP-secreting neurons are destroyed, while hereditary CDI accounts for approximately 1% of cases [21,22]. The primary pathogenesis involves impaired AVP synthesis or insufficient release from the posterior pituitary in response to osmotic stimuli [23].

Patients with CDI can be treated with vasopressin replacement therapy, which is effective in restoring water balance. In contrast, NDI, characterized by renal insensitivity to AVP, does not respond to exogenous hormone administration and currently lacks a definitive, targeted treatment [24]. Although the water deprivation test remains a key diagnostic tool for differentiating CDI from NDI, it was contraindicated in this patient due to the presence of severe hypernatremia, hyperosmolarity, and altered mental status. Plasma AVP measurement would have further aided the diagnosis, but it was unavailable at our facility. Given these limitations, the diagnosis of CDI relied on indirect clinical indicators. Notably, the patient’s urine specific gravity and clinical response to desmopressin therapy were pivotal. A reduction in urine specific gravity during treatment, in conjunction with symptomatic improvement following desmopressin administration, strongly supported the diagnosis of CDI [25]. Following treatment, the patient underwent pituitary MRI, which revealed a slightly heterogeneous pituitary signal with preserved size and morphology. No evidence of mass lesions, hemorrhage, or infarction was observed [26]. This imaging finding was critical in ruling out structural causes of acquired CDI. The typical MRI manifestation of CDI is the absence of the normal posterior pituitary “bright spot” on T1-weighted imaging, reflecting the depletion or dysfunction of AVP and its neurosecretory granules in the neurohypophysis [25]. However, in this case, a temporal or individual discrepancy may exist between the MRI findings and the patient’s hormonal functional status. Previous studies have shown that the visibility of the posterior pituitary high signal is not entirely equivalent to intact AVP function [27]. In certain CDI patients – particularly in the early stages of disease, with mild involvement, or in cases with obscure etiology (such as idiopathic or autoimmune CDI) – the structural integrity of the neurohypophysis may be partially preserved, and obvious radiologic abnormalities may not yet be apparent [28].

Based on these findings, it is proposed that the patient’s CDI may have resulted from diabetes mellitus-associated microvascular injury, which likely compromised blood supply to the hypothalamic–pituitary axis, thereby disrupting the synthesis and secretion of AVP. Furthermore, chronic systemic inflammation, a well-recognized feature of diabetes mellitus, may contribute to the development of complications such as hypernatremia and CDI [29]. Elevated inflammatory markers, including white blood cell count, C-reactive protein, and the neutrophil-to-lymphocyte ratio, are frequently observed in patients with type 2 diabetes mellitus and are associated with poor glycemic control and endothelial dysfunction. Emerging evidence suggests that inflammation may also impair AVP secretion and action, further disrupting water homeostasis and contributing to the pathogenesis of CDI [30]. Additionally, the uric acid to high-density lipoprotein cholesterol ratio, a composite marker of metabolic and inflammatory stress, has been linked to poorly controlled diabetes mellitus and its complications, suggesting a potential connection between metabolic inflammation and CDI [31]. Inflammatory responses may directly influence kidney function and AVP activity, playing a central role in the development of both hypernatremia and CDI in diabetic patients. These findings underscore the importance of comprehensive management strategies that target not only glycemic control but also systemic inflammation to prevent and mitigate complex complications such as CDI [32,33].

Severe hypernatremia, defined as a serum sodium concentration exceeding 160 mmol/L, and extreme hypernatremia (>190 mmol/L), are associated with significant morbidity and mortality rates exceeding 60% [34,35]. Hyperosmolarity causes cellular dehydration, particularly in neural tissues, leading to neurological dysfunction, coma, and death [36,37]. Extreme hypernatremia is rare, and reports describing electrocardiographic changes at such high sodium levels are limited [38]. Sodium, the principal extracellular cation, is critical for cellular depolarization and action potential generation. Hypernatremia-related electrocardiographic abnormalities, including QT prolongation and reduced P and QRS amplitudes, have been documented. Guidelines for chronic hypernatremia recommend a correction rate of 10–12 mmol/L/24 h to prevent cerebral edema [39]. However, recent studies suggest that slower correction rates in hospitalized patients with severe hypernatremia may be associated with increased mortality [40]. Although evidence on extreme hypernatremia is scarce, several case reports have demonstrated successful rapid correction under close monitoring without neurological complications [41]. Both overly rapid and excessively delayed correction carry significant risks [42]. Rapid correction may result in cerebral edema and myocardial ischemia, while insufficient correction can lead to persistent hypernatremia and its complications [43]. In the present case, a combination of fluid resuscitation and sodium-regulated continuous blood purification enabled safe normalization of serum sodium levels and reduction of urine output. The serum sodium decreased from 195.6 to 144.3 mmol/L over 79 h, corresponding to a correction rate of approximately 0.64 mmol/L/h. This rate was below the maximum recommended 2 mmol/L/h for acute hypernatremia to prevent cerebral edema but exceeded the conventional 10–12 mmol/L24 h target for chronic hypernatremia. Given the critical presentation, the accelerated correction was justified and achieved without neurological complications under continuous monitoring [44,45]. This case underscores the importance of carefully balancing the rate of sodium correction to prevent potentially fatal complications while ensuring effective management of hypernatremia.

## 4 Conclusions

In conclusion, this case demonstrates the complex interplay between CDI and HHS in a patient with poorly controlled type 1 diabetes mellitus. The occurrence of severe hypernatremia and ventricular fibrillation due to combined hypovolemia and electrolyte imbalance is exceptionally rare. This includes careful correction of electrolyte abnormalities, strict glycemic control, and hemodynamic stabilization. The case further emphasizes the importance of considering CDI in patients presenting with persistent polyuria and refractory hypernatremia, even after partial normalization of blood glucose levels. Additionally, it highlights the critical necessity of continuous cardiac monitoring during severe electrolyte disturbances.

## Supplementary Material

Supplementary Figure
