# Supplementary figures and images for: Hyperosmolar hyperglycemic state with severe hypernatremia coexisting with central diabetes insipidus: A case report and literature review
Source: Open Life Sci. 2025 Nov 6;20(1):20251194. doi: 10.1515/biol-2025-1194 (PMC12596032; doi:10.1515/biol-2025-1194)

## Supplementary material

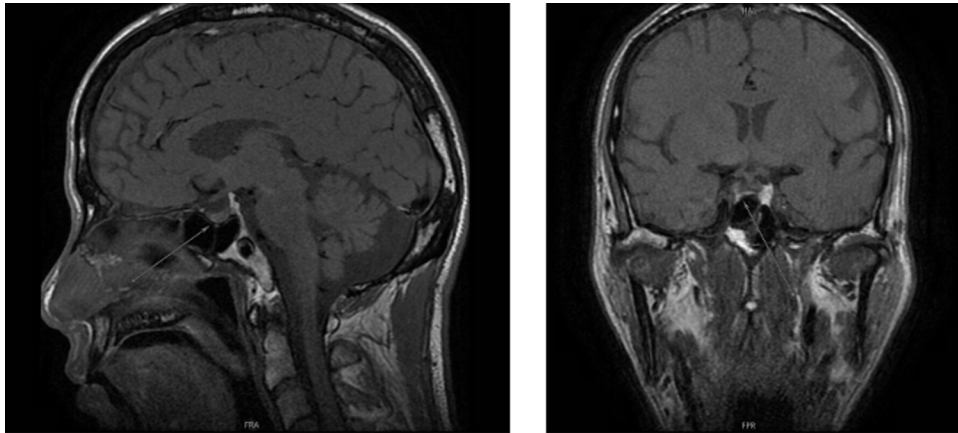

**Figure S1:** Magnetic Resonance Imaging (MRI) of the patient's head.

Supplement: Supplementary Figure [file biol-2025-1194-sm.pdf]
